# Using ultrasound guided needle biopsy in conjunction with GeneXpert MTB/RIF to diagnose epididymal tuberculosis

**DOI:** 10.1097/MD.0000000000036344

**Published:** 2023-12-29

**Authors:** Jia Cui, Xiuju Li, Yong Yu, Feng Xue, Jiakai Tian, Qinghu Yan

**Affiliations:** a Department of Ultrasound, Shandong Public Health Clinical Center, Shandong University Jinan, Shandong, China; b Department of Ultrasound Medicine, Shandong Provincial Hospital Affiliated to Shandong First Medical University, Jinan, China; c Department of Radiology, Qilu Hospital of Shandong University, Jinan, Shandong, China.

**Keywords:** acid-fast staining, GeneXpert MTB/RIF, Mycobacterium tuberculosis culture

## Abstract

This study aimed to investigate the diagnostic utility of percutaneous ultrasound-guided needle biopsy conjunction with GeneXpert MTB/RIF for epididymal tuberculosis. A retrospective analysis was conducted on the pathological and laboratory examinations of 20 patients with epididymal lesions undergoing ultrasound guided biopsy at Shandong Public Health Clinical Center. Laboratory examination included acid-fast staining, Mycobacterium tuberculosis culture by BACTEC MGIT 960, and GeneXpert MTB/RIF test. Diagnosis and complications were comprehensively analyzed. Among the 20 patients, 15 had epididymal tuberculosis and 5 had non-epididymal tuberculosis. Ten patients had granulomatous inflammation and necrotic tissues. The sensitivity and specificity of acid-fast staining, Mycobacterium tuberculosis culture, and GeneXpert MTB/RIF for the diagnosis of epididymis tuberculosis were 26.67% and 100.00%, 33.33% and 100.00%, and 86.67% and 100.00%, respectively. The diagnostic value analysis of the 3 detection techniques indicated that the GeneXpert MTB/RIF technique (Kappa = 0.765, Area under the curve (AUC) = 0.933) was superior to Mycobacterium tuberculosis culture (Kappa = 0.200, AUC = 0.667) and acid-fast staining (Kappa = 0.154, AUC = 0.633). Ultrasound-guided percutaneous biopsy is a safe procedure. The GeneXpert MTB/RIF test has high sensitivity, specificity, and superior AUC value, which are of great value in the diagnosis of epididymal tuberculosis and rifampicin resistance detection.

## 1. Introduction

Extrapulmonary tuberculosis accounts for 10% of all tuberculosis cases.^[1]^ Urogenital tuberculosis accounts for 30% to 40% of extrapulmonary tuberculosis cases, with epididymal tuberculosis being even more uncommon.^[2,3]^ Due to its insidious onset and mild symptoms, early epididymal tuberculosis can easily be missed.^[4]^ The epididymis can be divided into 3 sections: head, body, and tail. Furthermore, it primarily comprises epididymal tubules that migrate to the vas deferens of the tail. Mycobacterium tuberculosis can enter the prostate and seminal vesicles via urine and retrograde through the vas deferens, forming irregular masses in the epididymal tail. The epididymal body and head are subsequently invaded, causing the entire epididymis to swell, and the testis is finally invaded. This can also be caused by a primary infection transmitted through the bloodstream to the epididymis. Clinically, epididymal tuberculosis is clinically similar to testicular tumors, testicular torsion, and testicular bacterial inflammation, making it difficult to determine a definitive diagnosis under the present medical conditions.^[5,6]^ Ultrasound has the advantages of fast, simple, and no radiation, and it can be used for both the primary screening and advanced detection of epididymal tuberculosis.^[7]^ In the early stage of tuberculosis, the caseous lesions in the nodules are small, which mainly manifests as exudation and hyperplasia, and the blood flow signals are more abundant. If the infection develops further, necrosis forms in the lesion center and peripheral microvessels dilate, presenting abundant peripheral blood flow signals. As the lesion develops, the caseous necrosis area becomes enlarged and fused, most of the normal tissue structure is destroyed, and the lesion area presents with a decreased or no signal.

However, because the ultrasonic manifestations of epididymal tuberculosis are very similar to those of epididymal tumors, epididymal granuloma, and non specific epididymitis, pathological and microbial examinations are still required to confirm the definitive diagnosis.^[8]^ because of its real-time, accurate, and safe characteristics, ultrasound-guided puncture can be used for both pathological biopsy and laboratory examinations to confirm the diagnosis. Real-time fluorescence quantitative nucleic acid amplification for rifampin resistance (GeneXpert MTB/RIF) is a relatively novel tuberculosis diagnosis technology,^[9]^ which has higher sensitivity and specificity than traditional tuberculosis tests and can be used for rifampin resistance detection. This study aimed to determine the utility of ultrasound-guided percutaneous epididymal puncture in conjunction with GeneXpert MTB/RIF for the diagnosis of epididymal tuberculosis.

## 2. Materials and methods

### 2.1. Study subjects

Twenty patients with clinically suspected epididymal tuberculosis who were admitted to our hospital between March 2019 and March 2021 were enrolled in the present study. This study was approved by the ethics committee of Shandong Public Health Clinical Center. All methods were performed in accordance with relevant guidelines and regulations. All patients underwent pathological and laboratory examinations using ultrasound-guided puncture biopsy of the specimens. Clinical symptoms and ultrasound characteristics were then combined for comparison and analysis. According to the World Health Organization (WHO) guidelines^[10]^ and the Chinese Medical Association Tuberculosis Clinical Diagnostic Criteria,^[11]^ clinically diagnosed tuberculosis patients must meet the following criteria: clinical symptoms consistent with tuberculosis, clinical imaging highly suggestive of tuberculosis, and effective anti-tuberculosis treatment. All lesions were routinely examined using ultrasonography prior to needle biopsy. Patient information was retrieved from clinical records, including age, sex, comorbidities, laboratory tests, and response to treatment. None of these patients received anti-tuberculosis therapy prior to biopsy. This study was approved by the Ethics Committee of the Shandong Public Health Clinical Center (Shandong Chest Hospital) (2021XKYYEC-22). This study was conducted in accordance with the principles of the Declaration of Helsinki.

## 3. Ultrasound guided puncture

**Figure 1.**
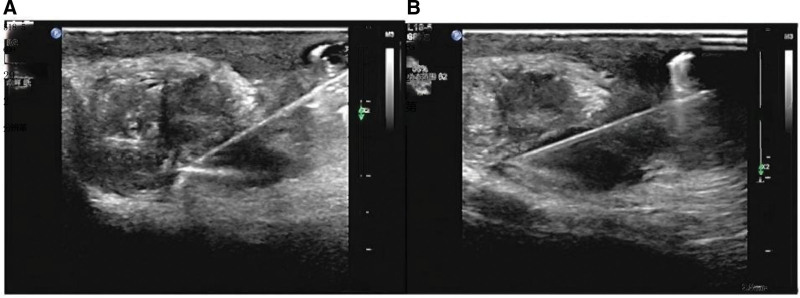
(A, B): The representative ultrasound imaging clearly reveals the hypoechoic nodules in the epididymis tail, local rupture and scrotum involvement. The ultrasound also clearly directed the puncture needle when performing the biopsy at the focus.

A Philips Q5 ultrasonic diagnostic instrument with a probe frequency of 7 to 18 MHz. All biopsies were collected under ultrasound guidance by the ultrasound surgeon who performed the punctures. Briefly, the patient was placed in a supine position according to the location of the lesion. The biopsy site was routinely disinfected, sterile toweled, and subjected to local layered anesthesia with 2% lidocaine hydrochloride. A semi-automatic cutting biopsy needle (18 G, 10 cm; BD, USA) was used to slowly puncture the lesion to 2 to 3 times under continuous real-time ultrasound monitoring. (Fig. 1). The biopsy specimens of all patients were subjected to pathological examination, acid-fast staining, BACTEC MGIT 960 Mycobacterium tuberculosis *c*ulture, and the GeneXpert MTB/RIF assay.

## 4. Pathological examination

The epididymal tissues were fixed with 10.0% formalin and embedded in paraffin before being sectioned sequentially. Sections with a thickness of 4 μm were stained with H&E and observed under a light microscope by 2 experienced pathologists. Under light microscopy, typical lesions of tuberculosis, such as epithelioid granulomas, Langhans giant cells, caseous necrosis, and other lesions, such as fibrosis and calcification fibrosis, were visible in the pathological tissue. This finding was consistent with the pathological characteristics of tuberculosis.

## 5. Pathogen tests

The acid-fast staining method (Zhuhai Basso, China) and modified alkaline compound red method were used according to the manufacturer instructions. The BACTEC MGIT 960 (BD, USA) Mycobacterium culture monitoring system and supporting reagents were used, and the strains were identified according to the manufacturer instructions. After adding an appropriate amount of phosphate buffer, the pathological tissues were homogenized using the micro-grinder FAST-PREP-24 (MP Biomeidcals, USA), which was subsequently used for GeneXpert MTB/RIF detection (Cepheid, USA),^[12]^ which is based on semi-nested polymerase chain reaction and automated fluorescence detection.^[13]^

## 6. Statistical analysis

All data were processed in Excel 2010 and statistically analyzed using SPSS 20.0. Normally distributed data were expressed as mean and standard deviation (X ± SD), whereas median and quartile spacing (M ± IQR) were used to express non-normally distributed data. The receiver operating characteristic (ROC) curve, area under the ROC curve (AUC), KAPPA value, sensitivity, specificity, positive predictive value, and negative predictive value were used for statistical analyses. Statistical significance was set at *P* < .05.

## 7. Results

### 7.1. General clinicopathological characteristics

The age of the patients ranged from 31 to 76 to years old, with a mean age of 46.3 ± 18.0 years old. The longest disease was 2 years, and the shortest duration was 3 months. Among the 15 cases of epididymal tuberculosis confirmed in the present study, 9 patients (60.0%) had coexisting tuberculosis of another organ. Furthermore, for these 15 cases, the clinical manifestations in 14 cases (93.3%) were scrotal mass or scrotal pain and falling sensation, while the clinical manifestations in the remaining cases (6.7%, 1/15) were fever and night sweats. There were No complications occurred in any of the 15 patients. However, 1 patient (6.7%, 1/15) had mild local bleeding, which resolved after the operation was stopped.

## 8. Ultrasonic manifestations

All 15 patients had varying degrees of epididymal changes: 11 patients had lesions in the tail; 3 patients had lesions in both the head and tail; and 1 patient had lesions in the head, body, and tail. Two-dimensional ultrasonography revealed that the lesions in the epididymis had a heterogeneous echo area. In 11 patients, the volume of the epididymis increased, the lesions were mainly in the tail, the boundary was unclear, the internal echo decreased, and the color Doppler results revealed a decrease in the blood flow signal. Six patients had scrotal swelling, thickening of the scrotal wall, irregular hypoecho in the scrotum, visible sinus tract, and significantly reduced or no blood flow signal in the color Doppler lesions. Furthermore, 5 patients presented with scrotal ulceration. In the 10 cases with testicular involvement, ultrasonography revealed a testicular mass with low blood flow signal. Yang et al^[14]^ considered that the decreased blood flow signal in the lesion was caused by caseous necrosis and calcification, which led to destruction of the vascular structure.

## 9. Pathological abnormalities

In the present study, ultrasonography of epididymal tuberculosis revealed a variety of pathological changes. Among the 15 patients, 10 had granulomatous inflammation and necrotic tissue, 2 had chronic inflammatory cells with necrotic tissue, and 3 had chronic inflammatory cell infiltration. Since these were detected in these patients relatively early, there was no evidence of calcification.

## 10. Laboratory results

**Figure 2.**
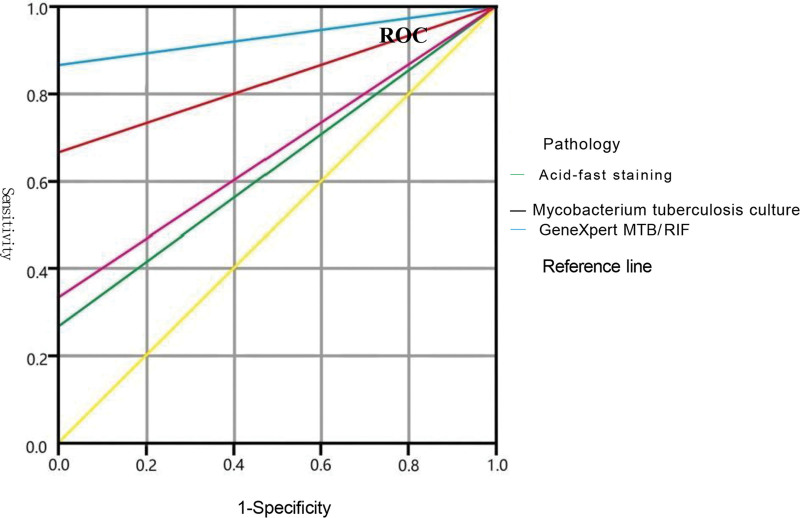
ROC curves of pathology, acid-fast staining, Mycobacterium tuberculosis culture, GeneXpert MTB/RIF and Reference line. ROC = receiver operating characteristic.

Among the 15 patients, 5 (33.33%, 5/15) were positive for the MGIT960 Mycobacterium tuberculosis culture, 4 (26.67%, 4/15) were positive for acid-fast staining, and 14 (86.67%, 13/15) were positive for the GeneXpert MTB/RIF assay, which had the highest positivity rate (Table 1). The specificity of acid-fast staining, tuberculosis culture, and GeneXpert MTB/RIF technique in the diagnosis of epididymis tuberculosis was 100.00%. In addition, all cases with a positive Mycobacterium tuberculosis culture were positive for the GeneXpert MTB/RIF assay. However, histopathological results were negative for the 5 confirmed patients. As shown in Table 2, GeneXpert MTB/RIF improved the pathogen detection rate in tissue biopsy specimens by 20.00% (3/15). The diagnostic values for epididymis tuberculosis were as follows: GeneXpert MTB/RIF technique (AUC = 0.933) > Mycobacterium tuberculosis culture (AUC = 0.667) > acid-fast staining (AUC = 0.633) (Table 2, Fig. 2). However, the KAPPA consistency test revealed that GeneXpert MTB/RIF had general consistency (KAPPA = 0.765), whereas acid-fast staining (KAPPA = 0.200) and tuberculosis culture (KAPPA = 0.154) had poor consistency (Table 2).

**Table 1:** Diagnostic results of pathology and the 3 laboratory methods for epididymal tuberculosis (n).

Detection technique	Detection result	Clinical comprehensive diagnosis		
		Epididymal tuberculosis	Non-epididymal tuberculosis	Total
Pathology	Positive	10	0	10
	Negative	5	5	10
	Total	15	5	20
Acid-fast staining	Positive	4	0	4
	Negative	11	5	16
	Total	15	5	20
Mycobacterium tuberculosis culture	Positive	5	0	5
	Negative	10	5	15
	Total	15	5	20
GeneXpert MTB/RIF	Positive	13	0	13
	Negative	2	5	7
	Total	15	5	20

**Table 2:** Diagnostic value of pathology and the 3 laboratory methods for epididymal tuberculosis.

Detection techniques	AUC	Sensitivity (%)	Specificity (%)	Kappa value	*P*	Jordan index
Pathology	0.833	66.67%	100.00%	0.500	.010	66.67%
Acid-fast staining	0.633	26.67%	100.00%	0.154	.197	26.67%
Mycobacterium tuberculosis culture	0.667	33.33%	100.00%	0.200	.136	33.33%
GeneXpert MTB/RIF	0.933	86.67%	100.00%	0.765	<.001	86.67%

## 11. Discussion

Pulmonary tuberculosis is mainly tuberculosis, and is mainly confirmed through respiratory tract specimens. For patients without respiratory symptoms, it is extremely difficult to diagnose extrapulmonary tuberculosis using common laboratory and imaging tests. Tuberculosis of the male reproductive system is a type of extrapulmonary tuberculosis that most commonly affects the epididymis, seminal vesicles, prostate, and testes. Owing to its rich blood supply, the caudal epididymis is the first to be affected by the most serious lesion. Because the vas deferens runs alongside the tail of the epididymis, Mycobacterium tuberculosis can easily spread directly along the vas deferens to the tail of the epididymis.^[15,16]^ Microscopically, multiple tuberculous granulomas of varying sizes can be observed in swollen epididymal tissues with extensive caseous necrosis, which are frequently accompanied by significant acute or chronic nonspecific inflammation. Kim et al reported that epididymal tuberculosis mostly occurs in the tail of the epididymis,^[17]^ while other studies revealed that epididymal tuberculosis mostly involves the whole epididymis or head.^[18]^ In the present study, tuberculosis lesions were mostly found in the caudal epididymis. It has been widely considered that epididymal tuberculosis is predominantly bilateral. In the present study, 8 cases (53.3%) were bilateral, while 7 cases (46.7%) were unilateral. Epididymal tuberculosis can be accompanied by pathological changes, such as granulation tissue formation, caseous necrosis and fibrosis, resulting in complex and diverse acoustic images.^[19]^ In the present group of cases, 10 cases with sinus formation all had testicular involvement.

Ultrasound is the first choice for epididymal imaging examination and can display the size, shape, structure, boundary, and other characteristics of the epididymis. Color Doppler ultrasound can reveal the distribution of blood flow in the epididymis. Ultrasonic guidance enables safe, effective, and accurate sampling while avoiding blood vessels and necrotic tissues, making it an ideal method for epididymal tuberculosis biopsy. In the present experiment, pathological features were obtained by ultrasound-guided puncture, and the samples were taken for laboratory examination. The diagnostic sensitivity, specificity, and AUC value of pathology were 66.67%, 100.00%, and 0.833, respectively, indicating a good diagnostic value. In terms of safety, ultrasound-guided biopsy is performed under direct vision, allowing for real-time monitoring of the location of the needle tip, observation of the blood supply of the lesion and necrosis, and avoidance of the blood vessels to ensure the safety of the puncture. In this study, the procedure was simple, repeatable, and had no complications.

Laboratory tests are necessary to confirm TB diagnosis. GeneXpert MTB/RIF is an automated polymerase chain reaction test that accurately detects Mycobacterium tuberculosis and its resistance to Rifampin.^[20]^ This can be used as an alternative test for the routine (including routine microscopy, culture, or histopathology) detection of specific non-respiratory specimens (lymph nodes and other tissues) in patients suspected of extrapulmonary tuberculosis. GeneXpert MTB/RIF is widely used in the diagnosis of extrapulmonary tuberculosis, but there are relatively few studies on epididymal tuberculosis. Some scholars^[21]^ have verified that the sensitivity of GeneXpert MTB/RIF in the diagnosis of bone and joint tuberculosis is 82% with a specificity of 100 00%. Some studies^[22]^ have confirmed that the sensitivity and specificity of the GeneXpert MTB/RIF method for pleural tuberculosis were 64 7%, 100.0%, respectively. Some studies^[23]^ have confirmed that the sensitivity of GeneXpert MTB/RIF for the diagnosis of urinary tuberculosis was 64%, and the specificity was 100.0%. The sensitivity and specificity of GeneXpert MTB/RIF in the diagnosis of brain tuberculosis were 23 0%, 100.0%.^[24]^ In this study, the sensitivity and specificity of GeneXpert MTB/RIF in the diagnosis of epididymal tuberculosis were 93.30% and 100%, respectively. respectively, which are consistent with the diagnostic results of other extrapulmonary tuberculosis cases. The sensitivity and specificity of GeneXpert MTB/RIF were higher than those of the other laboratory tests. The sensitivity, specificity, and AUC values of this study were higher because the lesions were relatively shallow, and more specimens were obtained. The difference in sensitivity and specificity at each site is mainly related to the different methods of specimen acquisition and different collection volumes. Compared to traditional acid-fast staining and tuberculosis culture technology, GeneXpert MTB/RIF is more valuable in the diagnosis of epididymal tuberculosis, which is beneficial for the early detection and timely guidance of clinical anti-tuberculosis drug treatment. In the present study, the proportional methods for detecting rifampin resistance in bacterial colonies obtained from 2 tuberculosis culture-positive cases were consistent with the results of the GeneXpert MTB/RIF method. The diagnostic value of GeneXpert MTB/RIF was also demonstrated through its superior AUC value when compared to other methods, although the test consistency was insufficient due to the rarity of epididymal tuberculosis and the small number of cases included in the present study.

Lack of research: 1. Although GeneXpert MTB/RIF can detect Mycobacterium tuberculosis and rifampicin resistance, it cannot replace the detection of rifampicin resistance. 2. The number of patients included in this study was not particularly large. 3. Most patients included in the study were clinically suspected of having tuberculosis, resulting in a specificity of 100%, which is consistent with many research results and does not affect its diffusion.

## 12. Conclusion

Ultrasound-guided percutaneous biopsy is safe. The expert mtb/rif test has high sensitivity, specificity, and superior AUC value, which are of great value in the diagnosis of epididymal tuberculosis and rifampicin resistance detection.

## Acknowledgments

The authors gratefully acknowledge the financial support of the Foundation of Shandong Health and Health Committee (project number 2019WS535). We thank Medjaden Inc. for scientific editing of this manuscript.

## Author contributions

**Data curation:** Jia Cui, Xiuju Li.

**Formal analysis:** Jia Cui, Xiuju Li, Feng Xue, Jiakai Tian.

**Funding acquisition:** Qinghu Yan.

**Investigation:** Yong Yu, Jiakai Tian.

**Methodology:** Jia Cui, Xiuju Li, Yong Yu, Feng Xue, Jiakai Tian, Qinghu Yan.

**Project administration:** Jia Cui, Qinghu Yan.

**Resources:** Jia Cui, Feng Xue.

**Validation:** Yong Yu.

**Writing – original draft:** Jia Cui.

**Writing – review & editing:** Qinghu Yan.
